# Preoperative assessment of breast volume to aid surgical planning: comparison of software-based mammographic measurements with subsequent mastectomy volumes

**DOI:** 10.1186/bcr3697

**Published:** 2014-11-03

**Authors:** I Teo, P Whelehan, EJ Macaskill, D Alex Munnoch, S Vinnicombe, Andy Evans

**Affiliations:** 1 NHS Tayside, Dundee, UK; 2 University of Dundee, UK

## Introduction

The proportion of breast volume excised during conservation surgery for breast cancer is crucial to cosmetic outcomes. Validated, expedient methods for accurate preoperative quantification of breast volume are lacking. This study evaluated breast volume measurements calculated by Volpara® breast density software, by comparing them with actual mastectomy volumes.

## Methods

From a prospective clinical database, 31 patients were identified for whom Volpara® (Matakina Technology Limited, New Zealand) volume measurements and mastectomy volumes were available. All patients had undergone skin-sparing mastectomy (SSM), bilateral in one case. Specimen volumes had been measured using a water-displacement technique. Volpara® volumes for the corresponding CC and MLO view of each of the 32 breasts were averaged and compared with the mastectomy volumes. Correlation was assessed using the Pearson correlation coefficient.

## Results

Volpara® breast volumes were, as expected, consistently higher than SSM volumes but with a very strong correlation (Pearson correlation coefficient for average Volpara® volumes and mastectomy volumes = 0.82 (*P *< 0.01)).

## Conclusion

The excellent correlation between Volpara® and SSM volumes suggests that this readily available and convenient preoperative measure of breast volume could be used as a tool to aid surgical planning in women with breast cancer, which might be particularly useful in those women not scheduled for preoperative MRI. When coupled with accurate measurement of tumour size plus required excision margin volume, possibly from three-dimensional ultrasound, this tool could potentially outperform current subjective preoperative methods of assessing relative volumes. Further validation work is needed.

